# Influence of Low-Level Prenatal Exposure to PCDD/Fs and PCBs on Empathizing, Systemizing and Autistic Traits: Results from the Duisburg Birth Cohort Study

**DOI:** 10.1371/journal.pone.0129906

**Published:** 2015-06-12

**Authors:** Nikola Nowack, Jürgen Wittsiepe, Monika Kasper-Sonnenberg, Michael Wilhelm, Axel Schölmerich

**Affiliations:** 1 Department of Developmental Psychology, Ruhr-University Bochum, Universitätsstraße 150, D-44801, Bochum, Germany; 2 Department of Hygiene, Social and Environmental Medicine, Ruhr-University Bochum, Universitätsstraße 150, D-44801, Bochum, Germany; Institute for Health & the Environment, UNITED STATES

## Abstract

**Background:**

Polychlorinated dibenzo-*p*-dioxins and dibenzofurans (PCDD/Fs) and polychlorinated biphenyls (PCBs) are assumed to act as endocrine disruptor chemicals. Prenatal exposure to these pollutants might influence fetal steroid hormone levels, which are thought to be related to sex-typical development and autistic traits.

**Objectives:**

We examined associations of prenatal levels of PCDD/Fs and PCBs with autism traits and sex-typical behaviour in childhood.

**Methods:**

We measured levels of PCDD/Fs and PCBs in maternal blood samples during pregnancy using gas chromatography/high-resolution mass spectrometry. Sex-typical behaviour was assessed at 9 years of age (n = 96) and autistic traits at 10 years of age using the Social Responsiveness Scale (SRS; n = 100). Multiple regression analyses were conducted to estimate the associations between prenatal exposure and outcome variables.

**Results:**

Blood concentrations (WHO_2005_-TEq) of ƩPCDD/Fs ranged from 2.93–46.45 pg/g _lipid base_ (median = 12.91 pg/g _lipid base_) and concentrations of ƩPCBs were in the range of 1.24–25.47 pg/g _lipid base_ (median = 6.85 pg/g _lipid base_) which is within the range of German background exposure. We found significant negative associations between PCDD/F levels in maternal blood and SRS scores in the whole group (β = -6.66, *p* < .05), in girls (β = -10.98, *p* < .05) and, in one SRS subscale, in boys (β = -6.86, *p* < .05). For PCB levels, associations with one SRS subscale were significant for the whole study group as were associations with two subscales in girls. We did not find significant associations between PCDD/F or PCB levels and sex-typical behaviour for either sex.

**Conclusions:**

In an earlier part of this study, prenatal exposure to PCDD/Fs and PCBs was found to be associated with lower testosterone levels, therefore, our findings are consistent with the idea that autism spectrum conditions are related to fetal androgen levels. Several possible mechanisms, through which PCDD/Fs and PCBs might influence autistic behaviour, are discussed.

## Introduction

Autism spectrum conditions (ASC) are defined by a triad of core symptoms from an early age: Abnormal social development and communication as well as restricted interests or stereotypical behaviour [1]. The prevalence of ASC is estimated at 0.5–1.5% [2–4]. Causal mechanisms in the development of ASC are unclear. Genetic factors such as copy-number variations, gene mutations or gene deletions are considered [5], as indicated by high concordance rates among twins and families. Notably, the prevalence of autism has increased over recent years, which cannot be fully explained by changes in diagnostic criteria and improved diagnosis [6]. Therefore, recent research has also focused on the role of environmental factors in the aetiology of autism, mainly air pollutants such as metals, solvents, nitrogen oxide and particulate matter [7–12] but also phthalates [13,14] and pesticides [15]. However, two other, closely related groups of persistent organic pollutants (POPs), namely polychlorinated biphenyls (PCBs) and polychlorinated dibenzo-*p*-dioxins and dibenzofurans (PCDD/Fs), have recently come into focus of autism research.

PCBs are a group of 209 congeners which differ with regard to the number and position of chlorine atoms on the two phenyl rings which form the basic structure. They have been synthesized and used as synthetic oils since 1929 until their production was ceased in the late 1970s. PCDD/Fs, also known as dioxins, are inadvertent by-products of synthetic processes, e.g. combustion. The toxic effects of PCDD/Fs are predominantly mediated by binding to the aryl hydrocarbon receptor (AhR), a ligand-activated transcription factor [16]. Because PCDD/F congeners differ with regard to their toxicity, the concept of toxicity equivalency factors (TEF) was developed and, in 2005, reevaluated by a World Health Organization (WHO)-expert committee (WHO_2005_-TEF [17]). The toxicity of PCDD/F congeners is ranked in relation to 2,3,7,8-tetrachlorodibenzo-*p*-dioxin (TCDD), the most toxic congener. Within the group of PCBs, two subgroups can be distinguished by structure and mechanism of action. The non- and mono-ortho chlorosubstituted PCBs, also called dioxin-like PCBs, are structurally and mechanistically similar to PCDD/Fs, therefore, the concept of WHO_2005_-TEF was also applied to this subgroup. In contrast, non-dioxin-like, or poly-ortho chlorosubstituted, PCBs do not act through binding to AhR but exert their effects through other mechanisms, such as gene induction [18]. In our study, toxicity equivalents (TEq) were used as markers for PCDD/Fs and dioxin-like PCBs.

Although exposure of the general population has decreased over the last years, especially prenatal exposure to PCDD/Fs and PCBs is still of concern because of their accumulative character and the increased vulnerability of the immature organism. In addition to the neurotoxic potential, PCDD/Fs and PCBs act as endocrine disruptor chemicals (EDCs [19]), which might affect the steroid hormone system [20,21]. Prenatal exposure to EDCs is considered especially harmful for the fetus and even low doses can lead to irreversibly organizational effects, as Myers and colleagues [22] point out. In a concise review, Landrigan [23] emphasizes that children are more vulnerable to environmental chemicals because they are proportionally more exposed to toxins than adults, less able to detoxify chemicals and are undergoing rapid development in the fetal and early postnatal period which can be easily disrupted.

So far, few studies have examined the association between PCDD/Fs and PCBs and ASC. Kim and colleagues [24] demonstrated less knowledge about and greater exposure to PCDDs and PCBs in mothers of children with ASC. In the Finnish Prenatal Study of Autism, Cheslack-Postava et al. [25] found higher odds ratios for ASC for children with PCB levels above the 90^th^ percentile of control values. Mitchell et al. [26] examined post-mortem brain samples of children with autism and other neurodevelopmental disorders and found elevated PCB levels in brains of subjects with 15q11-q13 duplication, which is one genetic factor in autism, but not in subjects with autism of unknown aetiology. Otake and coworkers [27] compared PCB levels in preserved samples of umbilical cords from typically developing children and those with an ASC but failed to show significant differences between the two groups. However, sample sizes were very small and contamination of cord samples could not be excluded. In a very recent study, Braun et al. [28] identified a variety of EDCs in blood or urine samples of pregnant women of the HOME study and related these to autistic behaviour at 4–5 years of age. Whereas polybrominated diphenyl ether (PBDE)-28 was associated with a greater amount of autistic behaviour, PBDE-85, PCB-178 and PFOA were linked to less autistic behaviour. In a Vietnamese study, Nishijo and coworkers [29] compared children who were highly TCDD-exposed from breast milk to those with mild exposure and found elevated autistic traits in the former group. However, this was only true for TCDD levels but not for total PCDD/F concentrations. Increased PCB levels were also related to less social behaviour in infant cynomolgus monkeys [30] whereas increased levels of TCDD were related to more exploration and proximity in social interaction in rhesus monkeys [31]. For a review on environmental contributions to the aetiology of autism, see [32].

There are several possible mechanisms through which PCDD/Fs and PCBs may affect fetal brain development [21]. The thyroid system has been of great interest because thyroid hormones are important for brain development during the fetal phase [33]; however, one review revealed inconsistencies between studies assessing the relationship between PCBs and thyroid hormones [34]. Interaction with the sex steroid system, namely estrogens and androgens, is another possible mechanism. During pregnancy, sex steroids are crucial for sexual differentiation of the brain. It is known from animal experiments that androgens, such as testosterone, produced prenatally, induce sexually differentiated brain structure and function [35–38]. Fetal androgens affect not only the anatomy of the brain [39–41] but also sexually dimorphic behaviour and cognition, e.g. sexual behaviour, aggression, play behaviour and visuospatial ability, in rodents and non-human primates [35,37,42,43]. In humans, there is an increasing body of evidence that brain differentiation, sex-typical behaviour and sexually dimorphic cognitive abilities may result from prenatal androgen exposure [44–47]. Prenatal exposure to PCDD/Fs and PCBs might induce alterations in sex-specific brain development. In a Dutch birth cohort study, sex-specific play behaviour at 7 years of age was measured [48]. In boys, higher prenatal PCB levels were associated with less masculine play whereas in girls, higher levels of PCBs were related to more masculine play. PCDD/F levels predicted more feminine play in both girls and boys. Winneke et al. [49] found higher PCB levels to be associated with more feminine play behaviour in boys and reduced feminine play behaviour in girls. Faass and colleagues [50] found prenatal exposition to PCBs and PBDEs in rats to affect gene expression in sexually dimorphic brain areas and female sexual behaviour. Prenatal PCB exposure was also associated with a reduction of sexual behaviour in female adult rats [51,52]. The latter finding also suggests that effects may be different for males and females. Exposure to PCBs is also thought to reduce male fertility [53]. Moreover, increased PCB concentrations were related to lower testosterone levels in the blood of Native American male adults [54] and to reduced testosterone levels in female neonates and reduced estradiol levels in male neonates [55].

Steroid hormones, especially fetal testosterone (fT), are not only implicated in the development of sexually dimorphic behaviour but also in the aetiology of autism (for a review, see [56]). The Extreme Male Brain theory (EMB) of autism [2,57] suggests that autism can be seen as an extreme form of the male brain, i.e. normal sex differences, e.g. in eye contact, narrow interests, analytical thinking, empathy, engagement in social relationships, are exaggerated in ASC. According to the Empathizing-Systemizing (E-S) theory [2], sex differences in cognition and behaviour, e.g. in play behaviour, empathy and spatial skills, are explained by two dimensions, empathizing and systemizing. Empathizing is defined as “the drive to identify another person’s emotions and thoughts, and to respond to these with an appropriate emotion” ([2] p. 248). Systemizing is “the drive to analyse the variables in a system, to derive the underlying rules” ([2] p. 248). Boys are assumed to be more drawn to systemize than girls whereas girls are supposed to be more likely to empathize. Individuals with an ASC are assumed to have an extreme male brain, i.e. superior systemizing but impaired empathizing [58–60]. Numerous studies support the idea of increased fT levels in autism [61–64].

The present study is part of a long-term birth cohort study, the Duisburg birth cohort study. It could be shown that environmental levels of PCDD/Fs and PCBs have decreased over the last years [65], therefore, the study was aimed at finding out whether adverse effects of prenatal exposure to POPs were still observable despite lower background levels. Whereas Wilhelm et al. [66] found no associations between internal PCDD/F or PCB exposure and thyroid hormone status nor neurological status in the first 24 months of life, effects of prenatal exposure to PCDD/Fs and PCBs could be shown on steroid hormone levels in cord blood samples from neonates [55] and in serum levels from 6 to 8-year old children [67]. Moreover, effects on sex-typical play behaviour [49] and on attention performance [68] were found. The current study was aimed to assess whether prenatal exposure to PCDD/Fs and PCBs was associated with sex-typical behaviour (empathizing and systemizing) as well as autistic behaviour as an extreme form of male-typical behaviour.

## Methods and Materials

### Ethic approval

The Duisburg birth cohort study was approved by the Ethics Committee of Medical Faculty of the Ruhr-University Bochum (registry no. 1478), follow-up was approved by the Ethics Committees of the Medical Faculty (registry no. 3486–09) and Faculty of Psychology (January 31^st^, 2011) of the Ruhr-University Bochum. The study was conducted in accordance with the ethical principles for medical research involving human subjects as defined by the Declaration of Helsinki. All parents and children gave written consent.

### Study area and participants

The study was conducted in Duisburg, Germany. Duisburg belongs to the Ruhr District, an industrial conurbation. A detailed description of the study area is given elsewhere [66].

Between September 2000 and October 2002, pregnant women living in a predefined area in and around Duisburg were recruited. Initially, 234 healthy mother-child pairs participated. For further detail on the study population, see [66]. Since 2007, regular follow-up studies have been conducted to examine child development. Between November 2010 and September 2011 n = 136 mothers were asked to complete the German version of the Empathy-Systemizing Quotient (EQ-SQ [58], German version: [69]) and n = 99 filled out the questionnaire which is a response rate of 72.7%. Questionnaires with more than two missing answers were excluded (n = 3), so the finale sample was n = 96 (52 boys). The children were aged 8–11 years (Mean [M] = 9.42; standard deviation [SD] = .36). In October 2011 we sent out the Social Responsiveness Scale (SRS [70,71], German version: [72]). Out of 133 invited parents, n = 100 filled out the questionnaire which is a response rate of 75.2%. No returned questionnaires had more than two missing answers. The children were 9–12 years old (M = 10.23; SD = .59) and 54 were boys. At follow-up, we included any mother-child pair who had filled out the EQ-SQ and/or the SRS, so the sample was n = 116.

### Collection and analysis of biological material

Maternal blood (50 mL, n = 226) was mainly taken between the 28^th^ and 42^nd^ week of pregnancy (median week 32). In 11 cases, blood samples were taken during the first two weeks after birth. All samples were stored at -20°C until analysis. Analysis was performed at the Department of Hygiene of the Ruhr-University Bochum using their established method and following regular quality assessment. Details on blood sampling and analytical procedures are given in [73]. Briefly, the method includes the following steps: (1) extraction of blood fat using organic solvents, (2) multiple column chromatography cleanup using modified silica gels and activated charcoal, and (3) instrumental determination with capillary gas chromatography and high-resolution mass spectrometry (HRCG/HRMS). PCDD/Fs and dioxin-like PCBs were summarized using the WHO_2005_-TEq concept [17] and concentrations were calculated on lipid base. Following two recent publications from the Duisburg birth cohort study [49,67], we used the WHO_2005_-TEq ƩPCDD/Fs, ƩPCBs and ∑PCDD/Fs+PCBs.

### Assessment of gender-specific cognitive style

The Empathy-Systemizing Quotient (EQ-SQ) is a parent report questionnaire, which comprises 55 items (27 EQ items, 28 SQ items). Following Auyeung et al. [58], we used the combined version of the EQ and SQ in order to ease administration and to avoid response bias, which could arise if one questionnaire was filled out before the other. Parents are asked to rate their child’s behaviour (e.g. “*My child gets upset at seeing others crying or in pain*”, “*My child enjoys arranging things precisely (e*.*g*. *flowers*, *books*, *music collections*”) on a 4-point Likert scale (“definitely agree” to “definitely disagree”).

Norm data is available for the original version of the EQ-SQ from a sample of 1256 school children and 265 children with an ASC diagnosis. There is no norm data for the German version which was used for the first time in this study.

Psychometric properties are reported as satisfactory for the original versions of the EQ and SQ [58].

Reliability: Internal consistency (Cronbach’s alpha) was .93 for the EQ-C and .78 for the SQ-C. Test-retest reliability (6 months) was good (EQ *r*_tt_ = .86, SQ *r*_tt_ = .84).

Validity: There are no validity studies for the child versions of the EQ and SQ.

### Assessment of autistic traits

The Social Responsiveness Scale (SRS [72,73]) is a 65-item parent or teacher report questionnaire that assesses reciprocal social behaviour and autistic traits in children and adolescents from 4 to 18 years. The SRS is used as a screening instrument for ASC which measures dimensional autistic traits, i.e. a quantitative rather than a categorical approach which also captures sub-clinical autistic behaviour. Parents are asked to rate the child's behaviour (e.g. “*Seems self-confident when interacting with others*”, „*Knows when he/she is too close to someone or invading someone's space*“) on a 4-point Likert scale (“never true”to “almost always true“) over the previous six months. Higher SRS scores indicate more severe social deficits. The SRS generates a total score as well as scores on the following five subscales: Social Awareness, Social Cognition, Social Communication, Social Motivation and Autistic Mannerisms. The SRS also generates an ASC score which rates the severity of symptoms compared to children with an ASC. Norm data of the original version is based on a sample of 1600 children; norm data of the German version is based on a sample of more than 1400 children from the general population. Additionally, norm data from a clinical ASC sample is available (n = 160 [73]). Psychometric properties of the SRS are reported as satisfactory to good [74–77].

Reliability: Internal consistency (Cronbach's alpha) was reported to be .91 to .93 in the general population and .97 in a clinical sample. Test-retest reliabilities (*r*_tt_ = .84 to *r*_tt_ = .88 in the normative sample, *r*_tt_ = .97 in the clinical sample) and interrater correlation (*r* = .76 in the normative sample, *r* = .95 in the clinical sample) are good.

Validity: The SRS shows moderate to strong correlations with the Autism Diagnostic Interview (ADI-R; *r* = .7 [76,77]), the Autism Diagnostic Observation Scale (*r* = .48 [76]) and the Social Communication Questionnaire (*r* = .58 [74]). It agrees highly (90%) with the ADI-R [78] and has good sensitivity (.75), specificity (.96) and area under the curve (.95 [79]).

### Statistical analysis

Statistical analysis was performed using the IBM SPSS Statistics 22 software. We used log (ln) transformed concentrations of WHO_2005_-TEq ƩPCDD/Fs, ƩPCBs and ƩPCDD/Fs+PCBs in maternal blood as proxy markers for prenatal exposure. Sex differences on the EQ-SQ and SRS were calculated using *t*-Tests. Correlations between POPs and outcome measures (SRS total and subscale scores, EQ and SQ) were calculated using Pearson’s correlations. Hierarchical multiple regression analyses were conducted to examine the association between prenatal exposure and outcome measures. Within-sex analyses were conducted to check for effects in either sex. Initially, potential confounding variables included sex of the child (male/female), age of the child (years), maternal age at parturition (years), German nationality (no/yes), maternal level of education (low/medium/high), length of gestation (weeks), older siblings (no/yes), younger siblings (no/yes), alcohol consumption (no/yes) or smoking (no/yes) during pregnancy, duration of breast-feeding and maternal IQ. For maternal IQ, the vocabulary subtest from the German Wechsler Adult Intelligence Scale [80] was employed. In the final regression model, we included covariates correlating with the outcome with a significance level of *p* < .2. The final regression models included the following covariates: maternal age at parturition, maternal level of education, age of the child, presence of older siblings, alcohol consumption and smoking during pregnancy and nationality. These covariates were included in the first stage of regression analysis using the enter method. In the second stage, exposure levels and sex were included using the stepwise method (entry criterion was *p* < .05, removal criterion was *p* >.1). In order to examine whether the effects of PCDD/Fs or PCBs were modified by sex, a second analysis was conducted including a sex * exposure interaction term in the second stage using the stepwise method. Due to missing values, the sample size was reduced to n = 80 for all outcome variables. Throughout the analyses, a significance level of *p* < .05 was considered to be statistically significant, *p* < .10 was considered marginally significant.

## Results

### Descriptive data for the follow-up sample

Characteristics for the follow-up sample are summarized in Table 1. Slightly more boys (53.4%) participated. Compared to the original sample described by Wilhelm and colleagues (2008), maternal age at birth was slightly higher (32.6 years vs. 31.2 years). The percentage of German families (88.8% vs. 88.9%) and the level of education (54.3 vs. 56.6 for the highest level) remained nearly the same. However, the proportion of maternal smoking or alcohol consumption was higher in our sample than in the original sample (16.4% vs. 12.7% for smoking; 12.1% vs. 11.4% for alcohol consumption). Maternal IQ, measured by the vocabulary subtest of the Wechsler Adult Intelligence Scale, was slightly higher in our sample (11.1) than normative values from a German calibration sample (10.0 [80]). We compared participants and non-participants with regard to POP levels and covariates (maternal age at parturition, maternal level of education, maternal IQ, number of older siblings, alcohol consumption and smoking during pregnancy, nationality and duration of breast-feeding). Participants had significantly higher PCB levels (M = 7.27, SD = 3.88) than non-participants (M = 5.84, SD = 3.12); *t*(218.687) = 3.067, *p* < .01, equal variances not assumed. Regarding PCDD/F levels, differences between participants and non-participants were marginally significant; *t*(214.87) = 1.883, *p* = .061, equal variances not assumed.

**Table 1:** Descriptive data of the follow-up sample (n = 116).

Characteristic	M ± SD	n (%)
Mothers		
Age at parturition (years)	32.64 ± 4.33	
Level of education		
Low		0 (0.0)
Medium		52 (44.8)
High		63 (54.3)
Other		1 (0.9)
Nationality (German)		103 (88.8)
Length of gestation (weeks)	39.4 ± 1.5	
Smoking during pregnancy (yes)		19 (16.4)
Alcohol consumption during pregnancy (yes)		14 (12.1)
Maternal IQ (Vocabulary score from WAIS)^a^	11.1 ± 2.8	
Children		
Age at completion of EQ-SQ (years)^b^	9.42 ± 0.36	
Age at completion of SRS (years)^c^	10.23 ± 0.59	
Sex (male)		62 (53.4)
Older siblings (yes)		37 (31.9)
Younger siblings (yes)		44 (37.9)

M = mean; SD = standard deviation; n = numbers; EQ-SQ = Empathy-Systemizing Quotient; SRS = Social Responsiveness Scale.

^**a**^n = 105.

^b^n = 96.

^c^n = 100.

With regard to covariates, we found significant differences in maternal age, maternal levels of education, maternal IQ, smoking during pregnancy and duration of breast-feeding. Mothers of participants were significantly older at birth that mothers of non-participants (M = 32.64, SD = 4.33 vs. M = 30.15, SD = 4.90); *t*(232) = 4.108, *p* < .001, had higher levels of education (M = 3.89, SD = 1.13 vs. M = 3.11, SD = 1.20); *t*(232) = 5.104, *p* < .001, and higher IQ (M = 11.05, SD = 2.78 vs. M = 9.71, SD = 3.43); *t*(204) = 3.073, *p* < .01, had smoked less during pregnancy (M = .33, SD = .86 vs. M = .75, SD = 1.27); *t*(206.584) = 3.015, *p* < .01, equal variances not assumed, and had breast-fed their children longer (M = 36.28, SD = 21.84 vs. M = 26.80, SD = 21.39); *t*(193) = 3.055, *p* < .01.

### Exposure

Table 2 presents the concentrations of ƩPCDD/Fs (GM = 13.56 pg/g _lipid base_, 95%-CI = 12.34–14.73), ƩPCBs (GM = 6.31 pg/g _lipid base_, 95%-CI = 5.69–6.97) and ƩPCDD/Fs+PCBs (GM = 20.10 pg/g _lipid base_, 95%-CI = 18.31–21.91) (WHO_2005_-TEq) in maternal blood samples. Blood levels of POPs in our sample were not significantly higher than in the original sample. All concentrations are within the range of German background exposure [74].

**Table 2:** Concentrations of ƩPCDD/Fs, ƩPCBs and ƩPCDD/Fs+PCBs (WHO_2005_-TEq [pg/g _lipid base_]) in maternal blood samples.

WHO_2005_-TEq [pg/g _lipid base_]		All			Boys				Girls			
	n	Med	GM	95%-CI	n	Med	GM	95%-CI	n	Med	GM	95%-CI
PCDD/F	116	12.91	13.56	12.34–14.73	62	12.79	13.23	11.79–15.06	54	13.74	13.94	12.26–15.73
PCB	116	6.85	6.31	5.69–6.97	62	6.63	6.30	5.56–7.21	54	7.08	6.32	5.36–7.29
PCDD/F+PCB	116	19.85	20.10	18.31–21.91	62	19.08	19.67	17.60–22.28	54	21.06	20.61	18.03–23.22

n = numbers; Med = median; GM = geometric mean; 95%-CI = 95% confidence interval of GM.

### Outcome

EQ-SQ scores and SRS scores, at age 8–10 and 9–11 respectively, are shown in Table 3. All outcome variables had acceptable skewness (skewness < 1) and were normally distributed. On average, girls (M = 39.45, SD = 6.26) scored higher on the EQ than boys (M = 33.48, SD = 8.27); *t*(93) = 3.979, *p* < .001, and boys (M = 27.92, SD = 6.19) score higher on the SQ than girls (M = 23.32, SD = 7.02); *t*(93) = 3.253, *p* < .01. Significant sex differences were also found on the SRS Social Awareness subscale; *t*(98) = 2.376, *p* < .05, and on the ASC score; *t*(98) = 2.171, *p* < .05, with boys displaying more autistic traits than girls. For the SRS total score, sex differences were marginally significant; *t*(98) = 1.902, *p* = .06, with boys (M = 51.70, SD = 9.51) scoring higher than girls (M = 47.93, SD = 10.30). In our sample, scores on the EQ-SQ were close to those found in the original English version [58]. The original versions of the EQ and SQ [58] showed small negative correlations which were no longer significant when typically developing children and children with an ASC were analyzed separately. In our sample, the EQ and SQ were not significantly correlated (*r* = .137, *p* > .05). The EQ was significantly negatively related to the SRS total score (*r* = -.331, *p* < .01), the Social Awareness subscale (*r* = -.395, *p* < .001), the Social Communication subscale (*r* = -.388, *p* < .001) and also marginally significantly related to the Social Cognition subscale (*r* = -.218, *p* = .052) whereas the SQ was not significantly correlated with any of the SRS scales.

**Table 3:** SRS total and subscale scores and EQ-SQ scores for the whole group and for each sex separately.

	All				Boys				Girls			
Variables	n	M	SD	Range	n	M	SD	Range	n	M	SD	Range
SRS total	100	49.97	10.01	25–70	54	51.70	9.51	25–70	46	47.93	10.30	25–65
SRS SA	100	51.96	9.69	32–71	54	54.04	8.93	33–71	46	49.52	10.07	32–69
SRS SC	100	51.05	8.82	36–74	54	51.69	9.17	36–74	46	50.30	8.43	36–65
SRS SCO	100	51.23	9.00	34–73	54	52.00	8.81	34–73	46	50.33	9.24	36–68
SRS SM	100	50.19	10.53	28–84	54	51.65	10.37	33–84	46	48.48	10.56	28–80
SRS AM	100	53.94	7.37	44–78	54	54.54	7.70	44–68	46	53.24	6.97	45–78
SRS ASC Score	100	28.91	3.28	25–41	54	29.56	3.62	25–41	46	28.15	2.68	25–34
EQ	96	36.22	7.97	15–52	52	33.48	8.27	15–52	44	39.45	6.26	23–50
SQ	96	25.81	6.94	9–42	52	27.92	6.19	13–42	44	23.32	7.02	9–38

M = mean; SD = standard deviation; n = numbers; SRS = Social Responsiveness Scale; SRS total = total score of the SRS; SRS SA = Social Awareness subscale score; SRS SC = Social Cognition subscale score; SRS SCO = Social Communication subscale score; SRS SM = Social Motivation subscale score; SRS AM = Autistic Mannerisms subscale score; SRS ASC Score = T-score compared to children with autism; EQ = Empathy Quotient; SQ = Systemizing Quotient.

### Regression analyses

We conducted Pearson correlations to check for correlations between POPs and SRS total and subscales and found that SRS total scores and scores on the Social Cognition subscale were significantly related to all POPs (e.g. *r* = -.283, *p* < .01 for Social Cognition and PCDD/Fs), Social Communication was related to PCDD/Fs (*r* = -.253, *p* < .05) and PCBs (*r* = -.242, *p* < .05) and SRS Autistic Mannerisms subscale was related to PCBs (*r* = -.226, *p* < .05) and PCDD/Fs+PCBs (*r* = -.202, *p* < .05). Marginally significant correlations were found between POPs and SRS Social Motivation subscale (e.g. *r* = -.186, *p* = .06 for PCDD/Fs) and PCDD/Fs and Autistic Mannerisms (*r* = -.183, *p* = .07) whereas no significant relationships were found for SRS Social Awareness subscale. In girls, significant relationships were found between all POPs and three subscales (Social Cognition, all *p* < .001, Social Communication, all *p* < .05, and Autistic Mannerisms, all *p* < .01). As presented in Table 4, WHO_2005_-TEq ƩPCDD/F levels in maternal blood were significantly negatively associated with scores on the SRS in the whole group (β = -6.66, 95%-CI = -11.88 –-1.44) and in girls (β = -10.98, 95%-CI = -19.43 –-2.53). Associations between PCB levels and SRS total scores were also marginally significant in girls (β = -6.07, 95%-CI = -13.17–1.02, *p* = .09) but not in boys (β = -1.63, 95%-CI = -8.33–5.07). Similarly, levels of PCDD/Fs+PCBs were significantly negatively correlated with SRS total scores in the whole group (β = -6.81, 95%-CI = -12.13 –-1.49) and in girls (β = -11.21, 95%-CI = -19.86 –-2.57). Stepwise regression analyses revealed that, in girls, POP levels were the only significant predictor whereas in boys, significant predictors for SRS total scores were the presence of older siblings and maternal smoking during pregnancy. The exposure-outcome modification by sex was also significant. Regression analysis revealed significant negative associations between all POPs and SRS Social Cognition subscale for girls (e.g. β = -7.04, 95%-CI = -13.22 –-.85 for PCDD/Fs) whereas in boys, the only significant predictor for this subscale was maternal education. On the Social Communication subscale, we found significant negative associations with PCDD/Fs and PCDD/F+PCBs in the whole group (β = -6.24, 95%-CI = -11.13 –-1.35 for PCDD/Fs) and in girls (β = -9.61, 95%-CI = -17.21 –-2.01 for PCDD/Fs). In boys, smoking during pregnancy was the only significant predictor whereas smoking and maternal education were significant predictors in the whole group for the Social Communication subscale. PCDD/F levels were significantly negatively associated with scores on the Social Motivation subscale in the whole group (β = -6.43, 95%-CI: -11.94 –-.91) and in boys (β = -6.86, 95%-CI = -13.67 –-.06), but not in girls (β = -4.98, 95%-CI = -13.67–3.72). In girls, age of the child was the only significant predictor. Scores on the Autistic Mannerisms subscale were also negatively associated with PCB levels in the whole group (β = -3.65, 95%-CI = -7.05 –-.24) and with all POPs in girls (e.g. β = -8.39, 95%-CI = 14.14 –-2.64 for PCDD/Fs). In boys, older siblings and nationality were significant predictors. On the EQ, we did not observe significant associations with either exposure measure; sex was the only significant predictor. Pearson correlations between PCDD/F levels and SQ scores were significant in boys (*r* = -.285, *p* < .05) but no longer when adjusted for confounding variables. We also found female gender, but not levels of PCBs or PCDD/Fs+PCBs, to be negatively associated with SQ scores. Maternal alcohol consumption during pregnancy was also a significant predictor for the SQ in the whole group and in boys.

**Table 4:** Results of the hierarchical regression analyses of associations between concentrations of WHO_2005_-TEq ƩPCDD/Fs, ƩPCBs and ΣPCDD/Fs+PCBs in maternal blood [pg/g lipid base], and SRS, EQ and SQ scores.

Exposure variable	All		Boys		Girls		Sex * exposure
	β (95% CI)	*p*	β (95% CI)	*p*	β (95% CI)	*p*	*p*
**SRS total** ^**a**^							
PCDD/F^**b**^	**-6.66 (-11.88, -1.44)**	< .05	-2.37 (-9.18, 4.45)	.48	**-10.98 (-19.43, -2.53)**	< .05	< .05
PCB^**b**^	-3.99 (-8.61, .64)	.09	-1.63 (-8.33, 5.07)	.62	-6.07 (-13.17, 1.02)	.09	< .05
PCDD/F+PCB^**b**^	**-6.81 (-12.13, -1.49)**	< .05	-2.22 (-9.20, 4.77)	.52	**-11.21 (-19.86, -2.57)**	< .05	< .05
**SRS SA** ^**a**^							
PCDD/F^**b**^	-1.18 (-6.47, 4.11)	.66	-2.91 (-9.81, 3.99)	.40	1.06 (-8.01, 10.13)	.81	< .05
PCB^**b**^	-.456 (-5.09, 4.17)	.85	-2.97 (-9.72, 3.78)	.38	.53 (-6.67, 7.72)	.83	.06
PCDD/F+PCB^**b**^	-1.06 (-6.48, 4.35)	.70	-3.06 (-10.12, 3.99)	-.38	.78 (-8.50, 10.06)	.87	< .05
**SRS SC** ^**a**^							
PCDD/F^**b**^	-1.53 (-5.87, 2.82)	.49	2.95 (-3.01, 8.91)	.32	**-7.04 (-13.22,-.85)**	< .05	.18
PCB^**b**^	-1.14 (-4.94, 2.66)	.55	2.81 (-3.03, 8.65)	.34	**-5.17 (10.14,-.20)**	< .05	.16
PCDD/F+PCB^**b**^	-1.80 (-6.24, 2.64)	.42	3.13 (-2.96, 9.22)	.30	**-8.29 (-14.43, -2.15)**	< .01	.19
**SRS SCO** ^**a**^							
PCDD/F^**b**^	**-6.24 (-11.13, -1.35)**	< .05	-3.28 (-10.14, 3.57)	.34	**-9.61 (-17.21, -2.01)**	< .05	.13
PCB^**b**^	-3.21 (-7.61, 1.19)	.15	-1.80 (-8.58, 4.98)	.59	-5.11 (-11.49, 1.28)	.11	.13
PCDD/F+PCB^**b**^	**-5.84 (-10.88,-.80)**	< .05	-2.90 (-9.94, 4.14)	.41	**-9.58 (-17.40, -1.77)**	< .05	.16
**SRS SM** ^**a**^							
PCDD/F^**b**^	**-6.43 (-11.94,-.911)**	< .05	**-6.86 (-13.67,-.062)**	< .05	-4.98 (-13.67, 3.72)	.25	.08
PCB^**b**^	-4.27 (-9.17, .63)	.09	-5.44 (-12.25, 1.37)	.11	-1.12 (-8.16, 5.92)	.75	.09
PCDD/F+PCB^**b**^	**-6.43 (-12.08,-.78)**	< .05	-6.739 (-13.74, .26)	.06	-3.90 (-12.88, 5.07)	.38	.09
**SRS AM** ^**a**^							
PCDD/F^**b**^	-2.06 (-6.06, 1.93)	.31	2.74 (-2.51, 7.99)	.30	**-8.39 (-14.14, -2.64)**	< .01	.13
PCB^**b**^	**-3.65 (-7.05,-.24)**	< .05	1.71 (-3.49, 6.91)	.51	**-7.86 (-12.14, -3.57)**	< .01	< .05
PCDD/F+PCB^**b**^	-3.01 (-7.06, 1.04)	.14	2.51 (-2.88, 7.91)	.35	**-9.72 (-15.35, -4.09)**	< .01	.12
**EQ** ^**a**^							
PCDD/F^**b**^	2.21 (-1.63, 6.05)	.26	1.95 (-5.03, 8.93)	.57	2.25 (-2.35, 6.84)	.33	< .001
PCB^**b**^	.74 (-2.81, 4.30)	.68	.51 (-6.35, 7.37)	.88	.725 (-3.36, 4.81)	.72	<.001
PCDD/F+PCB^**b**^	1.90 (-2.05, 5.85)	.34	1.57 (-5.60, 8.74)	.66	.903 (-3.380, 5.186)	.67	< .001
**SQ** ^**a**^							
PCDD/F^**b**^	1.43 (-2.02, 4.87)	.41	-.60 (-5.27, 4.07)	.79	3.23 (-2.42, 8.88)	.25	< .05
PCB^**b**^	.92 (-2.24, 4.09)	.56	-.77 (-3.79, 5.33)	.73	1.23 (-3.82, 6.27)	.62	< .05
PCDD/F+PCB^**b**^	1.38 (-2.15, 4.91)	.44	-.17 (-4.96, 4.62)	.94	2.81 (-3.00, 8.62)	.33	< .05

SRS = Social Responsiveness Scale; SRS total = total score of the SRS; SRS SA = Social Awareness subscale score; SRS SC = Social Cognition subscale score; SRS SCO = Social Communication subscale score; SRS SM = Social Motivation subscale score; SRS AM = Autistic Mannerisms subscale score; EQ = Empathy Quotient; SQ = Systemizing Quotient.

^**a**^Analyses of SRS scores included 41 boys and 39 girls. Analyses of EQ and SQ scores included 40 boys and 40 girls.

^**b**^log (ln) transformed concentrations of PCDD/Fs, PCBs and PCDD/Fs+PCBs in maternal blood [pg/g lipid base]. Regression models were adjusted for maternal age at parturition, maternal level of education, age of the child, presence of older siblings, alcohol consumption, smoking during pregnancy and nationality.

## Discussion

The current study examined the influence of prenatal exposure to endocrine-disrupting chemicals on sex-typical behaviour and autistic traits. Sex-typical behaviour was defined as empathizing and systemizing, according to the E-S theory of sex differences. We associated PCDD/F and PCB levels in maternal serum during pregnancy with scores on the EQ-SQ and the SRS in a German birth cohort study to assess whether prenatal exposure to these compounds might alter sex-specific behaviour and autistic behaviour as an exaggerated form of male-typical behaviour, as suggested by the EMB theory of autism [2].

We found autistic behaviour to be negatively associated with levels of PCDD/Fs and PCBs in maternal blood. This was somehow surprising as exposure to EDCs would have been expected to have reversed effects on autistic traits. To date, only a limited number of studies on the association between PCDD/F and PCB levels and autism have been published and results are contradictory. Cheslack-Postava and colleagues [25] found elevated odds ratios for autism in children who had PCB and DDE levels above the 90^th^ percentile of control values. Mitchell and colleagues [26] found increased PCB-95 levels in post-mortem brain samples of subjects with 15q11-q13 duplication, one known genetic basis for autism. Perinatal exposure to TCDD from breast milk was also linked to more autistic behaviour in a Vietnamese study whereas total PCDD/F concentrations were unrelated to autistic behaviour [29]. However, in a recent study, Braun et al. [28] found PCB-178 to be associated with less autistic behaviour. Additionally, Negishi and coworkers [31] investigated rhesus monkeys, prenatally exposed to TCDD, a prototype for PCDD/F, and found TCDD-exposed monkeys to show more visual exploration and more mutual proximity in a social interaction with a peer subject and less stereotypic behaviour than control monkeys. Deficits in social interaction and stereotypic behaviour alongside impaired communication form the triad upon which autism is diagnosed. Thus, monkeys in this study showed less autistic behaviour. In contrast, Nakagami et al. [30] demonstrated less social behaviour in PCB-exposed infant cynomolgus monkeys, i.e. less approach and proximity to the mother and fewer gazes at the mother’s face, and Schantz and colleagues [81] found that monkeys, prenatally exposed to TCDD, showed more dominance during a play situation. However, impaired mother-infant interaction as well as dominance in play behaviour cannot be directly equated with autism, therefore, it is unclear how these findings should be interpreted with regard to ASC.

The exact mechanisms how PCDD/Fs and PCBs are related to neurodevelopment are yet unknown, however, numerous possible explanations for an association between these POPs and autistic traits exist. Recent research has provided convincing evidence for a link between steroid hormones, i.e. androgens and estrogens, and autism. A number of studies have focused on the role of androgens, especially fT, in the aetiology of ASC. FT has been linked to sex-typical behaviour, e.g. eye contact [82], vocabulary size [83], mentalizing [84], quality of social relationships and restricted interests [85], empathy [86], systemizing [87], play behaviour [88] and visuo-spatial abilities [89] and also to autistic traits and ASC [61–64]. PCBs are assumed to have anti-androgenic effects [90,91] and PCBs as well as PCDD/Fs are related to lowered testosterone levels in adult men [54] as well as in female neonates, as shown in an earlier analysis of this sample [55]. Androgens bind to the androgen receptor (AR), a ligand-activated transcription factor which is coded for by the AR gene and belongs to the nuclear receptor superfamily. Notably, AR contains three functional domains, one of which, namely the terminal domain, has been of special interest. This terminal domain consists of a CAG trinucleotide repeat sequence of variable length, ranging from 11–30 repeats with a mean repeat length of about 20–22 repeats. The length of the CAG repeat is inversely related to AR activity as a transcription factor [92] and short CAG lengths are associated with increased prenatal sensitivity to androgens, as measured by second-to-fourth digit ratio, a proxy marker for fT [93]. In female rodents, PCBs were shown to down-regulate AR gene expression [94] and Björk and colleagues [95,96] have demonstrated that the effects of PCDD/Fs and PCBs on AR activity depend on its CAG repeat length. These findings show that exposure to PCDD/Fs and PCBs might also lead to altered gene expression. Furthermore, variations on the AR gene were found in females with autism [97]. Therefore, one possible explanation for our findings might be the interaction of PCDD/Fs and PCBs with the AR and their influence on fetal androgen levels which, in turn, seem to be related to ASC.

Another possible explanation for our results is that PCDD/Fs and PCBs might have estrogenic or anti-estrogenic effects. They are assumed to be xenoestrogens, which possibly mimic or block endogenous estrogens or interfere with estrogen signaling pathways [98]. Kester and colleagues [99,100] suggest that exposure to PCBs might increase circulating estradiol through inhibition of sulfotransferase, an enzyme which is responsible for estradiol inactivation. Therefore, exposure to xenoestrogens might lead to an imbalance of androgens and estrogens.

It is known that PCDD/F and, to some extent, PCB effects are mediated by binding to AhR which acts by heterodimerizing with the aryl hydrocarbon receptor nuclear translocator protein (ARNT). It is also suspected that AhR interacts with the estrogen receptors (ER) α and β [16,98,101]. PCB exposure is related to increased expression of ESR2, a gene encoding ER β, and up-regulation of CYP19, a gene coding for aromatase [102]. Aromatase belongs to the cytochrome P450 superfamily and is crucial for the aromatization of androgens into estrogens. Interestingly, in a genetic association study, Chakrabarti and colleagues identified variations in the ARNT2 gene, ESR1, ESR2 and also CYP19A1 to be associated with ASC diagnosis and/or autism traits in the general population [103].

Estrogens assumedly play an important role in the development of the cerebellum and promote dendritic growth of cerebellar Purkinje cells (PC [104]). Abnormalities in the cerebellum have been implicated in autism (for a review, see [105]) and PCBs have been reported to disrupt development of cerebellar PC in rodents [106]. Hence, we assume that the AhR as well as the ER are possible targets for PCDD/F and PCB action and that variations in the genes coding for these receptors are related to ASC.

Recently, Hu and colleagues [107,108] have proposed a new candidate gene for autism: the retinoic acid-related orphan receptor α (RORA), a ligand-dependent orphan nuclear hormone receptor which binds DNA of target genes and regulates expression of these genes in cooperation with co-regulator proteins. In subjects with autism, gene expression of RORA was reduced [108] as was expression of RORA protein in post-mortem samples of the cerebellum and frontal cortex [109]. It could be demonstrated that RORA regulates genes that have previously been associated with autism [110]. Moreover, androgens and estrogens regulate RORA with androgens decreasing RORA expression and estrogens up-regulating it [111]. RORA itself transcriptionally regulates aromatase. Therefore, reduction of RORA leads to a negative feedback mechanism resulting in further accumulation of testosterone and a decline of estradiol. These results support the notion of increased fT levels in ASC, as proposed by the EMB theory of autism [2]. In addition to these findings, Sarachana and Hu [112] found evidence for the involvement of AR and ERα in RORA gene expression. Hence, RORA provides a plausible target gene for EDCs, such as PCDD/Fs and PCBs, because these compounds seem capable of interacting with AR and, via AhR, with ER and have also been associated with an up-regulation of CYP19A1 [102].

It is important to keep in mind that exposure levels in our group were considerably lower than those found in other cohort studies. It is possible that the effects of low level exposure to POPs differ from those of higher doses, e.g. in rats, exposure to low levels of PCBs and TCDD facilitated spatial learning [113] whereas higher doses exhibit opposite effects [114]. Beneficial effects of PCBs were also found with regard to motor development in three-month-old infants [115] and attention and visual perception in five-year-old children [116]. Thus, low levels of POPs might alter autistic traits in a manner different from higher levels explaining the contradictory results found so far.

Contrary to what we expected, sex, but not PCDD/F or PCB levels, was the only significant predictor for sex-typical behaviour, i.e. empathizing and systemizing, in the regression analysis. In boys, but not in girls, PCDD/F levels were significantly correlated with SQ scores but the effects were no longer significant when adjusted for confounding variables. This was surprising, as these two cognitive dimensions are thought to be related to fT levels. To our knowledge, no studies have examined the influence of prenatal exposure to EDCs on empathy or systemizing, so far. We included empathizing and systemizing in our analyses because of their suggested association with autism. In our analyses, empathy was negatively associated with SRS total scores but also with two SRS subscale scores, Social Awareness and Social Communication, in the whole group. Within-sex analyses showed that the EQ was negatively correlated with SRS total scores, Social Awareness and Social Communication subscales scores in boys and with Social Communication subscale scores in girls. Surprisingly, in girls, the SQ was also negatively related to Social Communication subscale scores. The Social Awareness and Social Communication subscales measure a person’s ability to receive social cues, e.g. facial expressions, and to react appropriately to them, respectively. These aspects relate to the domains of social interaction and communication whereas the third domain of the autism triad, repetitive behaviour and/or narrow interests, was not related to the EQ or the SQ in our sample. Unlike the SRS, which is a validated tool in screening for autism, the EQ-SQ is a new questionnaire that has not been validated yet. As the EQ-SQ was translated from the English original version, it contains many reversed items with double negatives (e.g. no. 51 “My child is not bothered about knowing the exact timings of the day’s plans.”). In German language, double negatives are not common and might, therefore, be misunderstood. In an American sample, Wright and Skagerberg [117] used re-worded versions of the adult EQ and SQ questionnaires and found shorter response time, indicating more rapid understanding of the questionnaires, and higher reliability of the SQ. It is possible that especially the wording of the SQ leads to confusion and inaccurate results. Additional measures of empathy, e.g. the “Reading the Mind in the Eyes” test [118], and systemizing, e.g. the Embedded Figures Test [119], will be included at follow-up.

A particular strength of this study lies in its design as we assessed children at different points of time. We were able to measure exposure to EDCs prenatally and assess sex-typical and autistic behaviour in middle to late childhood. This is important because even though instruments in autism diagnosis have been improved and age at diagnosis, especially in classic autism, has been lowered, children with Asperger Syndrome or High Functioning Autism, both of which are considered ‘milder’ forms of autism, are often diagnosed first at school-age or even later. We would like to point out that, despite our small sample, the association between PCDD/F and PCB levels and SRS scores, which we found particularly in girls, were robust and that regression analysis revealed that POP levels were the only significant predictors for autistic behaviour in girls. By using a well-validated autism rating scale, we ensured that we were also able to capture sub-clinical autistic behaviour. In contrast to using the “gold standard” instruments in autism diagnostics, i.e. the ADOS or ADI-R which produce a dichotomous rating (“autistic” vs. “non-autistic”), this procedure takes into account that children without a clinical diagnosis of autism may differ with regard to the extent of autistic traits. None of our participants had an official diagnosis of autism. However, 5 children (5%, 4 boys, 1 girl) reached an SRS score of 65 or above which indicates a potential diagnosis of an ASC.

Several noteworthy limitations must be kept in mind. Due to loss at follow-up, our sample was small and mothers taking part were better-educated than the normal population. Therefore, our sample might not be representative of the general population. Due to ethical requirements, mothers received information about their exposure levels at an early stage. It is possible that this might have influenced parenting behaviour.

Here, we investigated exposure to PCDD/Fs and PCBs in maternal blood. Exposure to EDCs in breast milk might also influence sex-typical development and autistic behaviour, as found in the study by Nishijo and coworkers [29]. To account for these influences, we conducted separate regression analyses with concentrations of POPs in breast milk. However, we did not find significant associations between levels of either POP in breast milk and any of the outcome variables but the sample size was smaller and associations were generally in the same direction.

Moreover, we only relied on parent-report. Parent questionnaires provide useful information about a child’s behaviour in everyday life but might also be biased in either direction. As mentioned before, the EQ-SQ has not been validated yet and certain phrases, e.g. double negatives, might have led to confusion. Data on the EQ-SQ and SRS from a larger study group including children with an ASC will be obtained at a later point of time and will help to validate the EQ-SQ.

We cannot exclude the possibility that comorbid disorders, such as separation anxiety disorder, attention disorders or attachment disorders, might have influenced SRS scores. During the appointment, parents filled out a medical questionnaire that also contained the question whether the child had received a formal diagnosis of any psychiatric disorder. n = 2 children had a formal diagnosis of attention deficit/hyperactivity disorder. Exclusion of these children from the analysis did not produce different results. It is, however, possible that participants had comorbid disorders but diagnosis had not been made and thus could not have been taken into account.

## Conclusions

The present study examined the influence of maternal levels of PCDD/Fs and PCBs on sex-typical development (empathizing and systemizing) and autistic behaviour in middle to late childhood. We found strong associations between PCDD/F and PCB levels and SRS scores in girls and, partly, in boys. These findings are consistent with the EMB theory of autism, suggesting that fetal androgen levels are related to ASC. Causal conclusions cannot be drawn without caution as autism and also subclinical autistic behaviour are of complex, multifactorial origin and are very likely not attributable to a single cause. However, we point out several possible mechanisms through which PCDD/Fs and PCBs might influence autistic behaviour, i.e. via androgens and AR, estrogens and ER as well as a novel candidate gene for autism, RORA, which is differentially regulated by steroid hormones and their receptors and might be at risk of being disrupted by EDCs. It should be kept in mind that our results might be likely to result from a gene-environment interaction, therefore, the present study is merely a first step in investigating a possible relationship between organic pollutants and autistic behaviour and does not have any health implications at this point of time. Further analysis of epigenetic effects of prenatal exposure to PCDD/Fs and PCBs are under way in our lab. Additional information on how POPs act on the endocrine system is required.
